# (5S*,6R*,7R*)-6-Formyl-5-phenyl-7-propyl­perhydro­pyrazolo[1,2-*a*]pyrazol-1-one

**DOI:** 10.1107/S1600536810016764

**Published:** 2010-06-05

**Authors:** Jinlan Yu, Qingshan Zhang, Yanhong Liu, Yunzheng Li, Qinpei Wu

**Affiliations:** a School of Chemical Engineering and Environment, Beijing Institue of Technology, Beijing 100081, People’s Republic of China; b Technical Institute of Physics and Chemistry, Chinese Academy of Sciences, Beijing 100080, People’s Republic of China

## Abstract

The title compound, C_16_H_20_N_2_O_2_, was obtained by catalytic asymmetric cyclo­addition of *trans*-3-propyl­acrolein with 1-benzyl­idenepyrazolid-3-one betaine. There are two symmetry-independent mol­ecules in the asymmetric unit. In both mol­ecules, the two five-membered heterocyclic rings adopt envelope conformations.

## Related literature

For the biological activity of bicylic pyrazolidinone derivatives, see: Indelicato & Pasini (1988 ▶); Jungheim & Sigmund (1987 ▶). For synthetic methods of five-membered bicyclic heterocycles, see: Chen *et al.* (2006 ▶, 2007 ▶).
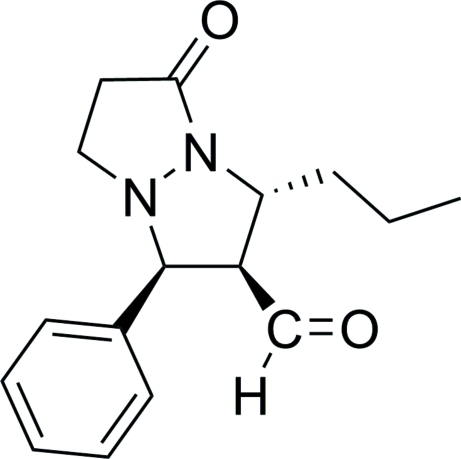


## Experimental

### 

#### Crystal data

C_16_H_20_N_2_O_2_
*M* *_r_* = 272.34
Triclinic, 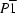

*a* = 8.557 (2) Å
*b* = 13.839 (3) Å
*c* = 13.905 (3) Å
α = 60.50 (3)°
β = 81.12 (3)°
γ = 81.22 (3)°
*V* = 1410.4 (7) Å^3^
*Z* = 4
Mo *K*α radiation
μ = 0.09 mm^−1^
*T* = 113 K
0.18 × 0.16 × 0.14 mm

#### Data collection

Rigaku Saturn CCD area-detector diffractometer
Absorption correction: multi-scan (*CrystalClear*; Rigaku/MSC, 2005 ▶) *T*_min_ = 0.985, *T*_max_ = 0.988
12841 measured reflections
6619 independent reflections
3540 reflections with *I* > 2σ(*I*)
*R*_int_ = 0.058

#### Refinement

*R*[*F* ^2^ > 2σ(*F* ^2^)] = 0.043
*wR*(*F* ^2^) = 0.112
*S* = 0.89
6619 reflections
365 parameters
H-atom parameters constrained
Δρ_max_ = 0.30 e Å^−3^
Δρ_min_ = −0.23 e Å^−3^

### 

Data collection: *CrystalClear* (Rigaku/MSC, 2005 ▶); cell refinement: *CrystalClear*; data reduction: *CrystalClear*; program(s) used to solve structure: *SHELXS97* (Sheldrick, 2008 ▶); program(s) used to refine structure: *SHELXL97* (Sheldrick, 2008 ▶); molecular graphics: *ORTEP-3* (Farrugia, 1997 ▶) and *SHELXTL* (Sheldrick, 2008 ▶); software used to prepare material for publication: *SHELXTL*.

## Supplementary Material

Crystal structure: contains datablocks global, I. DOI: 10.1107/S1600536810016764/lx2144sup1.cif

Structure factors: contains datablocks I. DOI: 10.1107/S1600536810016764/lx2144Isup2.hkl

Additional supplementary materials:  crystallographic information; 3D view; checkCIF report

## Figures and Tables

**Fig. 1.**
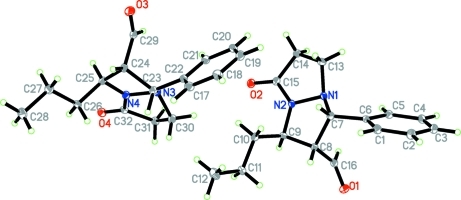
The molecular structure of the title compound with the atom numbering scheme. Displacement ellipsoids are drawn at the 30% probability level. H atoms are presented as a small cycles of arbitrary radius.

**Fig. 2.**
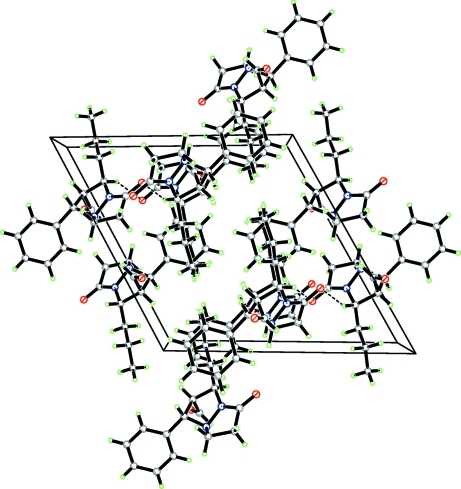
The crystal packing of the title compound, viewed along the a axis

## supplementary crystallographic information

### Comment

Bicylic pyrazolidinone derivatives are biologically active compounds, such as LY186826 exhibits high anti-bacterial activity (Jungheim *et al.* 1987; Indelicato *et al.* 1988). Recently, small organic molecules-catalysed asymmetric [3 + 2] dipolar cycloaddition of azomethine imines with alpha, beta-unsaturated aldehydes provides one of the most powerful strategies for the stereoselective synthesis of this five-membered bicyclic heterocycle (Chen *et al.*., 2006, 2007).). We observed that trifluoroacetic acid salt of 2-(pyrrolidinylmethyl)pyrrolidine catalyzes the synthesis of a series of bipyrazolidinone with excellent stereoselectivity.

We report the crystal structure of title compound, (2R,3R,4S)-2-propyl-3-formyl-4-phenyl-2,3,4,5- tetrahydropyrazolo [1,2-a] pyrazolidin-8-one (Fig. 1). There are two symmetry-independent molecules (A & B) in the asymmetric unit. In the title compound, two 5-membered heterocyclic rings adopt envelope conformation, and the configuration of the chiral centers, C7 (C23)S, C8 (C24)R and C9 (C25)R was assigned.

### Experimental

The trans-3-propyl-acrolein (39.3 mg, 0.4 mmol) was added to a mixture of 1-phenylidenepyrazolid-3-one (32.4 mg, 0.2 mmol), catalyst trifluoroacetic acid salt of 2-(pyrrolidinylmethyl)pyrrolidine (5.4 mg, 0.02 mmol) and water (6 muL) in THF (2.0 ml) at 10 °. the mixture was stirred at this temperature for 12 h. EtOAc (10 ml) was added, and the solution was washed with water, The organic phase was dried (Na_2_SO_4_), and concentrated under reduced pressure. The residue was isolated through short column chromatography on silica gel,which was eluded with EtOAc–petroleum to give the target compound(51.4 mg, 94%). 30 mg of the obtained product was dissolved in ethyl acetate (4 ml) and petroleum (1 ml) and the solution was kept at room temperature for 3 days to give colorless single crystals.

### Refinement

C—H were included in the riding model approximation with C—H distances 0.95–1.00 Å, and with U_iso_ = 1.2U_eq_ or 1.5U_eq_(methyl).

### Crystal data

**Table:**

C_16_H_20_N_2_O_2_	*Z* = 4
*M**_r_* = 272.34	*F*(000) = 584
Triclinic, *P*1	*D*_x_ = 1.283 Mg m^−^^3^
Hall symbol: -P 1	Mo *K*α radiation, λ = 0.71073 Å
*a* = 8.557 (2) Å	Cell parameters from 4363 reflections
*b* = 13.839 (3) Å	θ = 1.7–27.9°
*c* = 13.905 (3) Å	µ = 0.09 mm^−^^1^
α = 60.50 (3)°	*T* = 113 K
β = 81.12 (3)°	Block, colourless
γ = 81.22 (3)°	0.18 × 0.16 × 0.14 mm
*V* = 1410.4 (7) Å^3^	

### Data collection

**Table:**

Rigaku Saturn CCD area-detector diffractometer	6619 independent reflections
Radiation source: rotating anode	3540 reflections with *I* > 2σ(*I*)
confocal	*R*_int_ = 0.058
Detector resolution: 7.31 pixels mm^-1^	θ_max_ = 27.9°, θ_min_ = 1.7°
ω and φ scans	*h* = −11→10
Absorption correction: multi-scan (*CrystalClear*; Rigaku/MSC, 2005)	*k* = −18→17
*T*_min_ = 0.985, *T*_max_ = 0.988	*l* = −18→18
12841 measured reflections	

### Refinement

**Table:**

Refinement on *F*^2^	Secondary atom site location: difference Fourier map
Least-squares matrix: full	Hydrogen site location: inferred from neighbouring sites
*R*[*F*^2^ > 2σ(*F*^2^)] = 0.043	H-atom parameters constrained
*wR*(*F*^2^) = 0.112	*w* = 1/[σ^2^(*F*_o_^2^) + (0.039*P*)^2^] where *P* = (*F*_o_^2^ + 2*F*_c_^2^)/3
*S* = 0.89	(Δ/σ)_max_ = 0.001
6619 reflections	Δρ_max_ = 0.30 e Å^−^^3^
365 parameters	Δρ_min_ = −0.23 e Å^−^^3^
0 restraints	Extinction correction: *SHELXL97* (Sheldrick, 2008), Fc^*^=kFc[1+0.001xFc^2^λ^3^/sin(2θ)]^-1/4^
Primary atom site location: structure-invariant direct methods	Extinction coefficient: 0.032 (2)

### Special details

**Table:**

Geometry. All esds (except the esd in the dihedral angle between two l.s. planes) are estimated using the full covariance matrix. The cell esds are taken into account individually in the estimation of esds in distances, angles and torsion angles; correlations between esds in cell parameters are only used when they are defined by crystal symmetry. An approximate (isotropic) treatment of cell esds is used for estimating esds involving l.s. planes.
Refinement. Refinement of *F*^2^ against ALL reflections. The weighted *R*-factor wR and goodness of fit *S* are based on *F*^2^, conventional *R*-factors *R* are based on *F*, with *F* set to zero for negative *F*^2^. The threshold expression of *F*^2^ > σ(*F*^2^) is used only for calculating *R*-factors(gt) etc. and is not relevant to the choice of reflections for refinement. *R*-factors based on *F*^2^ are statistically about twice as large as those based on *F*, and *R*- factors based on ALL data will be even larger.

### Fractional atomic coordinates and isotropic or equivalent isotropic displacement parameters (Å^2^)

**Table:**

	*x*	*y*	*z*	*U*_iso_*/*U*_eq_	
O1	−0.36894 (19)	0.16690 (15)	0.55617 (16)	0.0407 (5)	
O2	0.24237 (17)	0.28648 (12)	0.22370 (13)	0.0234 (4)	
N1	0.1158 (2)	0.12298 (14)	0.50583 (15)	0.0182 (4)	
N2	0.1077 (2)	0.21896 (14)	0.39611 (15)	0.0183 (4)	
C1	−0.0489 (3)	0.11013 (18)	0.77713 (19)	0.0225 (5)	
H1	−0.0366	0.1841	0.7601	0.027*	
C2	−0.0993 (3)	0.03419 (19)	0.8840 (2)	0.0262 (5)	
H2	−0.1221	0.0562	0.9398	0.031*	
C3	−0.1168 (3)	−0.07422 (19)	0.9102 (2)	0.0254 (5)	
H3	−0.1509	−0.1267	0.9839	0.031*	
C4	−0.0848 (3)	−0.10551 (18)	0.82884 (19)	0.0231 (5)	
H4	−0.0969	−0.1797	0.8465	0.028*	
C5	−0.0344 (2)	−0.02851 (18)	0.72022 (19)	0.0205 (5)	
H5	−0.0127	−0.0506	0.6644	0.025*	
C6	−0.0159 (2)	0.07985 (17)	0.69382 (18)	0.0181 (5)	
C7	0.0306 (2)	0.16824 (17)	0.57711 (18)	0.0181 (4)	
H7	0.0978	0.2198	0.5799	0.022*	
C8	−0.1124 (2)	0.23707 (18)	0.50937 (18)	0.0182 (5)	
H8	−0.1631	0.2930	0.5330	0.022*	
C9	−0.0349 (2)	0.29584 (17)	0.38872 (18)	0.0177 (5)	
H9	−0.1057	0.2980	0.3367	0.021*	
C10	0.0068 (2)	0.41278 (17)	0.35206 (19)	0.0203 (5)	
H10A	0.0558	0.4121	0.4124	0.024*	
H10B	0.0859	0.4357	0.2865	0.024*	
C11	−0.1376 (3)	0.49711 (18)	0.3231 (2)	0.0233 (5)	
H11A	−0.2220	0.4689	0.3851	0.028*	
H11B	−0.1782	0.5047	0.2566	0.028*	
C12	−0.1025 (3)	0.61126 (19)	0.3005 (2)	0.0361 (6)	
H12A	−0.0638	0.6046	0.3665	0.054*	
H12B	−0.1997	0.6620	0.2829	0.054*	
H12C	−0.0213	0.6407	0.2377	0.054*	
C13	0.2890 (2)	0.09474 (19)	0.51073 (19)	0.0229 (5)	
H13A	0.3143	0.0149	0.5629	0.028*	
H13B	0.3364	0.1406	0.5333	0.028*	
C14	0.3474 (2)	0.12225 (18)	0.39071 (19)	0.0220 (5)	
H14A	0.4576	0.1439	0.3714	0.026*	
H14B	0.3424	0.0582	0.3784	0.026*	
C15	0.2308 (2)	0.22009 (18)	0.32376 (18)	0.0193 (5)	
C16	−0.2324 (3)	0.16164 (19)	0.52082 (19)	0.0247 (5)	
H16	−0.1977	0.1064	0.4986	0.030*	
O3	0.75718 (19)	0.66942 (15)	0.05764 (16)	0.0376 (5)	
O4	0.25782 (17)	0.78835 (12)	−0.27499 (13)	0.0240 (4)	
N3	0.3203 (2)	0.62734 (14)	0.00751 (15)	0.0185 (4)	
N4	0.3364 (2)	0.72338 (14)	−0.10197 (15)	0.0193 (4)	
C17	0.3464 (3)	0.60910 (19)	0.28130 (19)	0.0241 (5)	
H17	0.3053	0.6826	0.2658	0.029*	
C18	0.3752 (3)	0.5307 (2)	0.3887 (2)	0.0278 (6)	
H18	0.3545	0.5504	0.4462	0.033*	
C19	0.4346 (3)	0.42289 (19)	0.4124 (2)	0.0258 (5)	
H19	0.4535	0.3684	0.4863	0.031*	
C20	0.4659 (3)	0.39539 (19)	0.3285 (2)	0.0236 (5)	
H20	0.5075	0.3218	0.3446	0.028*	
C21	0.4371 (2)	0.47424 (18)	0.22027 (19)	0.0212 (5)	
H21	0.4589	0.4543	0.1629	0.025*	
C22	0.3764 (2)	0.58269 (18)	0.19570 (19)	0.0182 (5)	
C23	0.3467 (2)	0.67224 (18)	0.07920 (18)	0.0180 (5)	
H23	0.2526	0.7237	0.0820	0.022*	
C24	0.4902 (2)	0.74133 (18)	0.01171 (18)	0.0195 (5)	
H24	0.5023	0.7968	0.0358	0.023*	
C25	0.4435 (2)	0.80044 (17)	−0.10890 (18)	0.0183 (5)	
H25	0.5396	0.8023	−0.1607	0.022*	
C26	0.3621 (2)	0.91800 (17)	−0.14786 (19)	0.0200 (5)	
H26A	0.3151	0.9424	−0.2179	0.024*	
H26B	0.2744	0.9163	−0.0917	0.024*	
C27	0.4735 (3)	1.00255 (19)	−0.1668 (2)	0.0273 (5)	
H27A	0.5192	0.9790	−0.0965	0.033*	
H27B	0.5621	1.0036	−0.2222	0.033*	
C28	0.3917 (3)	1.11944 (18)	−0.2072 (2)	0.0310 (6)	
H28A	0.3469	1.1436	−0.2771	0.047*	
H28B	0.4690	1.1706	−0.2190	0.047*	
H28C	0.3063	1.1196	−0.1515	0.047*	
C29	0.6404 (3)	0.66558 (19)	0.02194 (19)	0.0258 (5)	
H29	0.6429	0.6113	−0.0013	0.031*	
C30	0.1594 (2)	0.5981 (2)	0.0128 (2)	0.0238 (5)	
H30A	0.0772	0.6439	0.0349	0.029*	
H30B	0.1480	0.5182	0.0651	0.029*	
C31	0.1497 (3)	0.62509 (18)	−0.10728 (19)	0.0226 (5)	
H31A	0.1936	0.5608	−0.1190	0.027*	
H31B	0.0388	0.6462	−0.1271	0.027*	
C32	0.2509 (3)	0.72308 (18)	−0.17447 (19)	0.0202 (5)	

### Atomic displacement parameters (Å^2^)

**Table:**

	*U*^11^	*U*^22^	*U*^33^	*U*^12^	*U*^13^	*U*^23^
O1	0.0188 (9)	0.0407 (12)	0.0407 (12)	−0.0039 (8)	0.0014 (8)	−0.0037 (9)
O2	0.0257 (9)	0.0238 (9)	0.0185 (9)	−0.0027 (7)	0.0015 (7)	−0.0093 (7)
N1	0.0192 (9)	0.0164 (10)	0.0148 (10)	0.0001 (8)	−0.0005 (8)	−0.0051 (7)
N2	0.0186 (9)	0.0161 (10)	0.0147 (10)	−0.0001 (7)	0.0003 (8)	−0.0041 (7)
C1	0.0258 (12)	0.0205 (12)	0.0215 (13)	0.0000 (9)	−0.0036 (10)	−0.0106 (10)
C2	0.0286 (13)	0.0319 (14)	0.0196 (13)	−0.0005 (10)	−0.0018 (10)	−0.0142 (11)
C3	0.0237 (12)	0.0272 (13)	0.0185 (12)	−0.0025 (10)	−0.0006 (10)	−0.0061 (10)
C4	0.0221 (12)	0.0188 (12)	0.0237 (13)	−0.0019 (9)	−0.0036 (10)	−0.0062 (10)
C5	0.0217 (12)	0.0197 (12)	0.0206 (12)	0.0001 (9)	−0.0033 (10)	−0.0101 (10)
C6	0.0144 (11)	0.0195 (12)	0.0192 (12)	0.0015 (9)	−0.0047 (9)	−0.0082 (9)
C7	0.0205 (11)	0.0169 (11)	0.0173 (12)	−0.0025 (9)	−0.0018 (9)	−0.0082 (9)
C8	0.0183 (11)	0.0172 (11)	0.0159 (11)	0.0004 (9)	−0.0001 (9)	−0.0064 (9)
C9	0.0157 (11)	0.0174 (11)	0.0186 (12)	0.0008 (9)	−0.0020 (9)	−0.0081 (9)
C10	0.0195 (11)	0.0198 (12)	0.0210 (12)	−0.0031 (9)	0.0001 (9)	−0.0094 (9)
C11	0.0246 (12)	0.0185 (12)	0.0225 (13)	−0.0004 (10)	−0.0030 (10)	−0.0068 (10)
C12	0.0433 (16)	0.0225 (14)	0.0423 (17)	0.0022 (11)	−0.0065 (13)	−0.0162 (12)
C13	0.0188 (11)	0.0244 (13)	0.0231 (13)	0.0015 (9)	−0.0035 (10)	−0.0100 (10)
C14	0.0186 (11)	0.0205 (12)	0.0255 (13)	−0.0004 (9)	0.0016 (10)	−0.0115 (10)
C15	0.0201 (11)	0.0194 (12)	0.0199 (13)	−0.0037 (9)	0.0008 (9)	−0.0108 (10)
C16	0.0197 (12)	0.0240 (13)	0.0207 (13)	−0.0032 (10)	−0.0055 (10)	−0.0022 (10)
O3	0.0236 (9)	0.0385 (11)	0.0368 (11)	−0.0081 (8)	−0.0111 (8)	−0.0039 (8)
O4	0.0277 (9)	0.0255 (9)	0.0189 (9)	−0.0003 (7)	−0.0049 (7)	−0.0105 (7)
N3	0.0213 (10)	0.0181 (10)	0.0148 (10)	−0.0049 (8)	−0.0023 (8)	−0.0059 (7)
N4	0.0208 (10)	0.0199 (10)	0.0149 (10)	−0.0029 (8)	−0.0030 (8)	−0.0059 (8)
C17	0.0243 (12)	0.0252 (13)	0.0227 (13)	0.0016 (10)	−0.0029 (10)	−0.0122 (10)
C18	0.0302 (13)	0.0331 (14)	0.0196 (13)	0.0004 (11)	−0.0022 (11)	−0.0131 (11)
C19	0.0255 (12)	0.0267 (13)	0.0204 (13)	−0.0050 (10)	−0.0033 (10)	−0.0065 (10)
C20	0.0204 (12)	0.0211 (12)	0.0256 (13)	−0.0017 (9)	−0.0053 (10)	−0.0075 (10)
C21	0.0218 (12)	0.0211 (12)	0.0210 (12)	−0.0032 (9)	−0.0016 (10)	−0.0101 (10)
C22	0.0156 (10)	0.0204 (12)	0.0195 (12)	−0.0039 (9)	−0.0017 (9)	−0.0097 (9)
C23	0.0182 (11)	0.0200 (12)	0.0171 (12)	−0.0016 (9)	−0.0017 (9)	−0.0099 (9)
C24	0.0212 (11)	0.0180 (12)	0.0167 (12)	−0.0044 (9)	−0.0016 (9)	−0.0055 (9)
C25	0.0173 (11)	0.0221 (12)	0.0168 (12)	−0.0039 (9)	−0.0010 (9)	−0.0099 (9)
C26	0.0188 (11)	0.0188 (12)	0.0202 (12)	0.0010 (9)	−0.0030 (9)	−0.0082 (9)
C27	0.0260 (13)	0.0242 (13)	0.0295 (14)	−0.0024 (10)	−0.0054 (11)	−0.0104 (11)
C28	0.0386 (15)	0.0221 (13)	0.0335 (15)	−0.0028 (11)	−0.0036 (12)	−0.0140 (11)
C29	0.0190 (12)	0.0253 (13)	0.0216 (13)	−0.0050 (10)	−0.0024 (10)	−0.0014 (10)
C30	0.0209 (12)	0.0280 (13)	0.0229 (13)	−0.0061 (10)	−0.0039 (10)	−0.0109 (10)
C31	0.0236 (12)	0.0212 (12)	0.0245 (13)	−0.0034 (10)	−0.0078 (10)	−0.0101 (10)
C32	0.0201 (11)	0.0204 (12)	0.0214 (13)	0.0027 (9)	−0.0051 (10)	−0.0115 (10)

### Geometric parameters (Å, °)

**Table:**

O1—C16	1.201 (3)	O3—C29	1.201 (3)
O2—C15	1.231 (3)	O4—C32	1.233 (3)
N1—N2	1.451 (2)	N3—N4	1.450 (2)
N1—C7	1.468 (3)	N3—C23	1.468 (3)
N1—C13	1.478 (3)	N3—C30	1.475 (3)
N2—C15	1.336 (3)	N4—C32	1.336 (3)
N2—C9	1.472 (3)	N4—C25	1.468 (3)
C1—C2	1.379 (3)	C17—C18	1.380 (3)
C1—C6	1.392 (3)	C17—C22	1.386 (3)
C1—H1	0.9500	C17—H17	0.9500
C2—C3	1.386 (3)	C18—C19	1.389 (3)
C2—H2	0.9500	C18—H18	0.9500
C3—C4	1.375 (3)	C19—C20	1.373 (3)
C3—H3	0.9500	C19—H19	0.9500
C4—C5	1.399 (3)	C20—C21	1.390 (3)
C4—H4	0.9500	C20—H20	0.9500
C5—C6	1.386 (3)	C21—C22	1.397 (3)
C5—H5	0.9500	C21—H21	0.9500
C6—C7	1.511 (3)	C22—C23	1.509 (3)
C7—C8	1.552 (3)	C23—C24	1.556 (3)
C7—H7	1.0000	C23—H23	1.0000
C8—C16	1.511 (3)	C24—C29	1.507 (3)
C8—C9	1.547 (3)	C24—C25	1.548 (3)
C8—H8	1.0000	C24—H24	1.0000
C9—C10	1.521 (3)	C25—C26	1.526 (3)
C9—H9	1.0000	C25—H25	1.0000
C10—C11	1.517 (3)	C26—C27	1.519 (3)
C10—H10A	0.9900	C26—H26A	0.9900
C10—H10B	0.9900	C26—H26B	0.9900
C11—C12	1.521 (3)	C27—C28	1.518 (3)
C11—H11A	0.9900	C27—H27A	0.9900
C11—H11B	0.9900	C27—H27B	0.9900
C12—H12A	0.9800	C28—H28A	0.9800
C12—H12B	0.9800	C28—H28B	0.9800
C12—H12C	0.9800	C28—H28C	0.9800
C13—C14	1.534 (3)	C29—H29	0.9500
C13—H13A	0.9900	C30—C31	1.533 (3)
C13—H13B	0.9900	C30—H30A	0.9900
C14—C15	1.526 (3)	C30—H30B	0.9900
C14—H14A	0.9900	C31—C32	1.524 (3)
C14—H14B	0.9900	C31—H31A	0.9900
C16—H16	0.9500	C31—H31B	0.9900
			
N2—N1—C7	102.06 (15)	N4—N3—C23	102.11 (16)
N2—N1—C13	101.67 (16)	N4—N3—C30	102.18 (16)
C7—N1—C13	118.14 (17)	C23—N3—C30	118.16 (17)
C15—N2—N1	114.00 (17)	C32—N4—N3	113.52 (18)
C15—N2—C9	133.46 (19)	C32—N4—C25	133.84 (19)
N1—N2—C9	112.53 (16)	N3—N4—C25	112.64 (17)
C2—C1—C6	120.9 (2)	C18—C17—C22	121.2 (2)
C2—C1—H1	119.5	C18—C17—H17	119.4
C6—C1—H1	119.5	C22—C17—H17	119.4
C1—C2—C3	120.2 (2)	C17—C18—C19	119.8 (2)
C1—C2—H2	119.9	C17—C18—H18	120.1
C3—C2—H2	119.9	C19—C18—H18	120.1
C4—C3—C2	119.7 (2)	C20—C19—C18	119.7 (2)
C4—C3—H3	120.2	C20—C19—H19	120.1
C2—C3—H3	120.2	C18—C19—H19	120.1
C3—C4—C5	120.3 (2)	C19—C20—C21	120.6 (2)
C3—C4—H4	119.9	C19—C20—H20	119.7
C5—C4—H4	119.9	C21—C20—H20	119.7
C6—C5—C4	120.2 (2)	C20—C21—C22	120.1 (2)
C6—C5—H5	119.9	C20—C21—H21	119.9
C4—C5—H5	119.9	C22—C21—H21	119.9
C5—C6—C1	118.8 (2)	C17—C22—C21	118.5 (2)
C5—C6—C7	122.7 (2)	C17—C22—C23	119.19 (19)
C1—C6—C7	118.45 (19)	C21—C22—C23	122.3 (2)
N1—C7—C6	113.67 (17)	N3—C23—C22	113.02 (18)
N1—C7—C8	100.75 (17)	N3—C23—C24	100.72 (17)
C6—C7—C8	114.04 (18)	C22—C23—C24	114.75 (18)
N1—C7—H7	109.3	N3—C23—H23	109.3
C6—C7—H7	109.3	C22—C23—H23	109.3
C8—C7—H7	109.3	C24—C23—H23	109.3
C16—C8—C9	110.58 (18)	C29—C24—C25	110.15 (18)
C16—C8—C7	110.91 (18)	C29—C24—C23	110.62 (18)
C9—C8—C7	103.10 (16)	C25—C24—C23	102.79 (17)
C16—C8—H8	110.7	C29—C24—H24	111.0
C9—C8—H8	110.7	C25—C24—H24	111.0
C7—C8—H8	110.7	C23—C24—H24	111.0
N2—C9—C10	111.97 (17)	N4—C25—C26	111.88 (17)
N2—C9—C8	100.87 (16)	N4—C25—C24	100.99 (17)
C10—C9—C8	113.29 (18)	C26—C25—C24	114.60 (17)
N2—C9—H9	110.1	N4—C25—H25	109.7
C10—C9—H9	110.1	C26—C25—H25	109.7
C8—C9—H9	110.1	C24—C25—H25	109.7
C11—C10—C9	112.23 (18)	C27—C26—C25	113.39 (18)
C11—C10—H10A	109.2	C27—C26—H26A	108.9
C9—C10—H10A	109.2	C25—C26—H26A	108.9
C11—C10—H10B	109.2	C27—C26—H26B	108.9
C9—C10—H10B	109.2	C25—C26—H26B	108.9
H10A—C10—H10B	107.9	H26A—C26—H26B	107.7
C10—C11—C12	113.0 (2)	C28—C27—C26	112.88 (19)
C10—C11—H11A	109.0	C28—C27—H27A	109.0
C12—C11—H11A	109.0	C26—C27—H27A	109.0
C10—C11—H11B	109.0	C28—C27—H27B	109.0
C12—C11—H11B	109.0	C26—C27—H27B	109.0
H11A—C11—H11B	107.8	H27A—C27—H27B	107.8
C11—C12—H12A	109.5	C27—C28—H28A	109.5
C11—C12—H12B	109.5	C27—C28—H28B	109.5
H12A—C12—H12B	109.5	H28A—C28—H28B	109.5
C11—C12—H12C	109.5	C27—C28—H28C	109.5
H12A—C12—H12C	109.5	H28A—C28—H28C	109.5
H12B—C12—H12C	109.5	H28B—C28—H28C	109.5
N1—C13—C14	102.53 (17)	O3—C29—C24	124.2 (2)
N1—C13—H13A	111.3	O3—C29—H29	117.9
C14—C13—H13A	111.3	C24—C29—H29	117.9
N1—C13—H13B	111.3	N3—C30—C31	101.93 (18)
C14—C13—H13B	111.3	N3—C30—H30A	111.4
H13A—C13—H13B	109.2	C31—C30—H30A	111.4
C15—C14—C13	102.67 (17)	N3—C30—H30B	111.4
C15—C14—H14A	111.2	C31—C30—H30B	111.4
C13—C14—H14A	111.2	H30A—C30—H30B	109.2
C15—C14—H14B	111.2	C32—C31—C30	103.10 (18)
C13—C14—H14B	111.2	C32—C31—H31A	111.1
H14A—C14—H14B	109.1	C30—C31—H31A	111.1
O2—C15—N2	125.6 (2)	C32—C31—H31B	111.1
O2—C15—C14	128.6 (2)	C30—C31—H31B	111.1
N2—C15—C14	105.74 (19)	H31A—C31—H31B	109.1
O1—C16—C8	124.3 (2)	O4—C32—N4	125.8 (2)
O1—C16—H16	117.9	O4—C32—C31	128.5 (2)
C8—C16—H16	117.9	N4—C32—C31	105.67 (19)
			
C7—N1—N2—C15	150.02 (17)	C23—N3—N4—C32	−150.98 (17)
C13—N1—N2—C15	27.6 (2)	C30—N3—N4—C32	−28.3 (2)
C7—N1—N2—C9	−29.2 (2)	C23—N3—N4—C25	28.8 (2)
C13—N1—N2—C9	−151.60 (16)	C30—N3—N4—C25	151.51 (16)
C6—C1—C2—C3	0.4 (3)	C22—C17—C18—C19	−0.3 (4)
C1—C2—C3—C4	−0.4 (3)	C17—C18—C19—C20	0.7 (4)
C2—C3—C4—C5	0.1 (3)	C18—C19—C20—C21	−0.6 (3)
C3—C4—C5—C6	0.2 (3)	C19—C20—C21—C22	0.2 (3)
C4—C5—C6—C1	−0.2 (3)	C18—C17—C22—C21	−0.1 (3)
C4—C5—C6—C7	−177.06 (19)	C18—C17—C22—C23	−178.5 (2)
C2—C1—C6—C5	−0.1 (3)	C20—C21—C22—C17	0.2 (3)
C2—C1—C6—C7	176.88 (19)	C20—C21—C22—C23	178.49 (19)
N2—N1—C7—C6	165.37 (16)	N4—N3—C23—C22	−165.83 (16)
C13—N1—C7—C6	−84.2 (2)	C30—N3—C23—C22	83.1 (2)
N2—N1—C7—C8	42.96 (19)	N4—N3—C23—C24	−42.91 (18)
C13—N1—C7—C8	153.36 (18)	C30—N3—C23—C24	−153.98 (17)
C5—C6—C7—N1	−23.8 (3)	C17—C22—C23—N3	−156.73 (19)
C1—C6—C7—N1	159.38 (19)	C21—C22—C23—N3	25.0 (3)
C5—C6—C7—C8	91.0 (2)	C17—C22—C23—C24	88.5 (2)
C1—C6—C7—C8	−85.9 (2)	C21—C22—C23—C24	−89.7 (2)
N1—C7—C8—C16	75.4 (2)	N3—C23—C24—C29	−74.4 (2)
C6—C7—C8—C16	−46.8 (2)	C22—C23—C24—C29	47.3 (2)
N1—C7—C8—C9	−42.98 (19)	N3—C23—C24—C25	43.14 (19)
C6—C7—C8—C9	−165.13 (17)	C22—C23—C24—C25	164.84 (17)
C15—N2—C9—C10	−56.6 (3)	C32—N4—C25—C26	56.3 (3)
N1—N2—C9—C10	122.41 (19)	N3—N4—C25—C26	−123.46 (19)
C15—N2—C9—C8	−177.3 (2)	C32—N4—C25—C24	178.6 (2)
N1—N2—C9—C8	1.6 (2)	N3—N4—C25—C24	−1.1 (2)
C16—C8—C9—N2	−93.61 (19)	C29—C24—C25—N4	92.5 (2)
C7—C8—C9—N2	25.0 (2)	C23—C24—C25—N4	−25.39 (19)
C16—C8—C9—C10	146.57 (18)	C29—C24—C25—C26	−147.07 (19)
C7—C8—C9—C10	−94.83 (19)	C23—C24—C25—C26	95.0 (2)
N2—C9—C10—C11	169.09 (18)	N4—C25—C26—C27	−174.41 (18)
C8—C9—C10—C11	−77.6 (2)	C24—C25—C26—C27	71.4 (2)
C9—C10—C11—C12	172.6 (2)	C25—C26—C27—C28	179.12 (19)
N2—N1—C13—C14	−35.4 (2)	C25—C24—C29—O3	125.5 (2)
C7—N1—C13—C14	−146.05 (18)	C23—C24—C29—O3	−121.5 (2)
N1—C13—C14—C15	32.4 (2)	N4—N3—C30—C31	35.74 (19)
N1—N2—C15—O2	172.3 (2)	C23—N3—C30—C31	146.78 (18)
C9—N2—C15—O2	−8.8 (4)	N3—C30—C31—C32	−32.3 (2)
N1—N2—C15—C14	−6.5 (2)	N3—N4—C32—O4	−171.5 (2)
C9—N2—C15—C14	172.5 (2)	C25—N4—C32—O4	8.7 (4)
C13—C14—C15—O2	164.7 (2)	N3—N4—C32—C31	7.1 (2)
C13—C14—C15—N2	−16.6 (2)	C25—N4—C32—C31	−172.7 (2)
C9—C8—C16—O1	−124.8 (2)	C30—C31—C32—O4	−165.3 (2)
C7—C8—C16—O1	121.5 (2)	C30—C31—C32—N4	16.2 (2)

## References

[bb1] Chen, W., Du, W., Duan, Y. Zh., Wu, Y., Yang, Sh. Y. & Chen, Y. Ch. (2007). *Angew. Chem. Int. Ed.* **46**, 7667–7670. 10.1002/anie.200702618 17768751

[bb2] Chen, W., Yuan, X. H., Li, R., Du, W., Wu, Y., Ding, L. Sh. & Chen, Y. Ch. (2006). *Adv. Synth. Catal.* **348**, 1818–1822.

[bb3] Farrugia, L. J. (1997). *J. Appl. Cryst.* **30**, 565.

[bb4] Indelicato, J. M. & Pasini, C. E. (1988). *J. Med. Chem.* **31**, 1227–1230. 10.1021/jm00401a026 3373491

[bb5] Jungheim, L. N. & Sigmund, S. K. (1987). *J. Org. Chem.* **52**, 4007–4013.

[bb6] Rigaku/MSC (2005). *CrystalClear* Rigaku/MSC Inc., The Woodlands, Texas, USA.

[bb7] Sheldrick, G. M. (2008). *Acta Cryst.* A**64**, 112–122. 10.1107/S0108767307043930 18156677

